# Optimising the induction of inflammation within preterm infant-derived intestinal epithelial organoids

**DOI:** 10.1038/s44355-026-00054-2

**Published:** 2026-02-02

**Authors:** Jonathan A. Chapman, Andrew M. Frey, Maria Emilia Dueñas, Jeremy M. Palmer, Andrea C. Masi, Nicholas D. Embleton, Matthias Trost, Janet E. Berrington, Christopher J. Stewart

**Affiliations:** 1 Newcastle University, Translational and Clinical Research Institute, Newcastle upon Tyne, UK; 2 Newcastle University, Biosciences Institute, Newcastle upon Tyne, UK; 3 Wesfarmers Centre of Vaccines and Infectious Diseases, The Kids Research Institute Australia, Perth Children’s Hospital, Nedlands, WA Australia; 4 Newcastle Neonatal Service, Newcastle upon Tyne Hospitals NHS Foundation Trust, Newcastle upon Tyne, UK; 5 Newcastle University, Population Health Sciences Institute, Newcastle upon Tyne, UK

**Keywords:** Inflammation, Gastrointestinal diseases, Gastrointestinal system, Intestinal stem cells

## Abstract

Preterm infants born <32 weeks gestation have abnormal microbial colonisation and dysregulated inflammation within the gut. Preterm infant-derived intestinal organoids (PIOs) represent a valuable model for investigating gut microbiome-host interactions and inflammatory responses. We optimised an inflammation model in PIO monolayers incubated within an anaerobic co-culture system that recreates the physiological oxygen gradient of the intestinal epithelium. We trialled multiple stimuli, including live and heat-killed pathobiont consortia, lipopolysaccharide (LPS) and flagellin. We found that a combination of apical LPS and basolateral flagellin, incubated for 3 h, elicited the most robust response. This was characterised by enhanced pro-inflammatory cytokine secretion, the potential for chemokine-driven immune recruitment, TNFα and IL17C pathway signalling, shifts from NF-κB to AP-1-mediated responses, and signs of tissue remodelling. This provides a framework for appropriate study design to disentangle the impacts of microbiome-host interactions in health and disease using intestinal organoids.

## Introduction

The development of organoid models to study human physiology has progressed rapidly during the last decade^1–4^. These ex vivo cultures have the advantage of recreating the cell type diversity of complex human tissues, while retaining the genetic and immune profiles of the host from which they are derived^5,6^. This gives organoids multiple advantages over traditional cancer-derived cell lines, which are not patient specific, contain only one cell type and may have differing physiologies from non-cancerous “healthy” cells. Intestinal epithelial organoids are derived from LGR5^+^ stem cells, found within intestinal crypts^7,8^. The crypt structures can be extracted from intestinal tissue and embedded within a hydrogel, such as Matrigel, where they grow as 3D spheroids^6^. These spheroids are naturally polarised, with an inner apical surface corresponding to the gut lumen and an outer basolateral surface that would interface with the lamina propria in vivo. Experiments can be performed using these 3D structures or the spheroids can be dissociated and seeded onto a support structure, such as a Transwell, to form a polarised 2D monolayer. These monolayers can be cultured statically, or incorporated into microfluidic systems, featuring a constant fluid flow, such as the cells would be subjected to in the gut^9,10^. Once confluent, organoid cultures can be differentiated to create a monolayer that contains the major cell types of the gut epithelium, namely enterocytes, goblet cells and enteroendocrine cells^6,8^. Being derived from crypt stem cells does however mean that such organoids lack immune cells, vasculature and neurons.

Preterm infants are born <37 weeks gestation and can be subdivided into moderate to late preterm (32 to <37 weeks), very preterm (28 to <32 weeks) and extremely preterm (<28 weeks). The bodily systems of very and extremely preterm infants are physiologically immature, meaning these babies are especially vulnerable to certain pathologies with high risks of morbidity and mortality, such as necrotising enterocolitis (NEC) and late onset sepsis (LOS)^11^. In the case of the gut, this takes the form of an intestinal epithelial barrier that is more permeable or “leaky”, due to its immaturity^12,13^. The human gut is a major site of microbial colonisation, and so the barrier function of the intestinal epithelium is critical for infection prevention, establishing immune toleration and maintaining host health^14^. Accordingly, some preterm infant pathologies have been linked to aberrant gut microbiome-host interactions and inflammation in the gut, following microbial colonisation after birth^15–20^. Consequently, there is strong interest in studying these interactions to uncover pathological mechanisms and develop new preventative therapies. Preterm infant-derived intestinal organoids (PIOs) are one such model system with which to do this. They are also vital for studying this specific population, as evidence indicates that PIOs exhibit distinct transcriptomes compared to adult-derived organoids, especially during exposure to live microbes^21,22^. Life stage of a patient therefore impacts organoid function.

To advance studies of gut microbiome-host interactions and epithelial immune responses in preterm infants, we present the optimisation of a model of inflammation within PIOs. Experiments were conducted using an anaerobic co-culture system that recreates the physiological oxygen gradient of the intestinal epithelium^23^. Different stimuli to induce inflammation were trialled and the host responses to each were characterised in depth. This work provides a framework for stimulating inflammation in intestinal organoids to facilitate novel approaches to dampen the pro-inflammatory response and promote gut health.

## Results

### Bacterial-derived components and a cocktail of live pathobionts induce distinct immune responses in organoids, which are impacted by incubation time

**Fig. 1:**
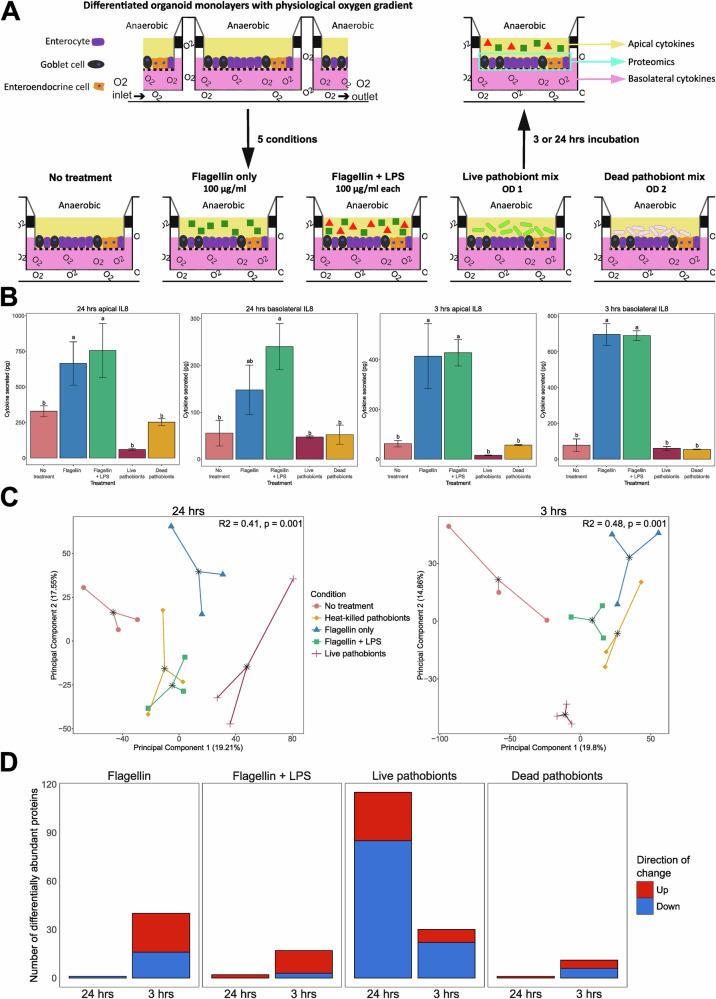
Comparisons of the effects of incubating preterm infant-derived intestinal epithelial organoids (PIOs) with purified bacterial stimuli (flagellin (100 μg/ml) and/or lipopolysaccharide (LPS) (100 μg/ml)), a cocktail of live bacterial pathobionts, or heat-killed bacterial pathobionts, for either 24 h or 3 h. **A** Schematic of PIO inflammation assays. **B** Bar plots showing the changes in both apical and basolateral IL8 secretion from PIOs, for both the 24 h and 3 h incubations. Within each plot, conditions with the same letters are not significantly different. **C** Principal component analysis of PIO proteomics data for both the 24 h and 3 h incubations. Groups are coloured by condition. **D** Per condition comparison of the number of differentially abundant proteins identified within PIO proteomics data for both incubation durations. Thresholds for differential abundance were set at adjusted *p* ≤ 0.05 and log2(foldchange) ≥ +/− 0.58 (real term fold change of +/−1.5).

We first compared the inflammatory effects of incubating PIOs with purified bacterial stimuli (flagellin and/or LPS), a cocktail of live bacterial pathobionts, or heat-killed bacterial pathobionts (Fig. 1A). This pathobiont cocktail contained nine species (Supplementary Table 1), all of which were cultured from preterm infant stool samples and collectively represent the top nine most abundant pathobionts detected in the preterm gut^24^. These differing stimuli were added to the apical side of PIO monolayers, to simulate their presence in the gut lumen. We first tested 24 h incubation as per previous intestinal-derived organoid work^21,23,25–27^, followed by 3 h, which was the middle value of shorter 2–4 h incubation times that have been employed in other stimulation experiments with epithelial cells^25,28–31^.

Our initial focus for characterising the response of PIOs to treatment was quantification of IL8 secretion. IL8 is a generic and highly abundant marker of inflammation throughout the body^32,33^, including in inflammatory gut pathologies impacting both infants and adults^34–36^. An increase in its secretion was therefore considered an important component of the inflammatory phenotype we aimed to induce in our model. Following 24 h incubation, apical IL8 secretion was significantly increased from PIOs treated with flagellin (*p* = 0.038) and flagellin + LPS (*p* = 0.0097), compared to the no treatment control (Fig. 1B). Flagellin + LPS also induced a significant increase in basolateral IL8 (*p* = 0.0014; Fig. 1B). Neither apical nor basolateral IL8 secretion from PIOs treated with the live or heat-killed pathobiont cocktails were significantly different from the no treatment control (both *p* > 0.05; Fig. 1B). The same pattern of IL8 secretion was observed when the experiment was repeated with a 3 h incubation time (Fig. 1B), with the exception that treatment with flagellin alone also induced an increase in basolateral IL8 (*p* < 0.001).

Proteomics was performed on PIO cell lysates from both the 24 h and 3 h experiments. For both datasets, unsupervised ordinations clustered by treatment (24 h, *p* = 0.001; 3 h, *p* = 0.001) (Fig. 1C). Comparing differentially abundant proteins (DAPs) between each treatment and the no treatment control at 24 h showed the live pathobiont cocktail had the greatest impact (total of 115 DAPs; 30 upregulated and 85 downregulated) (Fig. 1D). The other three conditions all had <5 DAPs compared to the no treatment control (Fig. 1D). Conversely, the 3 h incubation time reduced the number of DAPs within PIOs treated with the live pathobionts (115 at 24 h to 30 at 3 h), while a larger number of DAPs were identified for the other three conditions (Fig. 1D). Removing the 1.5-fold change threshold for differential abundance increased the number of DAPs in all but one condition highlighting the presence of a large number of small-scale fold changes within the dataset (Supplementary Fig. 1; Supplementary Table 2). However, the increases in DAPs were heavily concentrated within the flagellin treatment at 3 h and the live pathobiont treatment at 24 h. Overall, the 3 h experiment contained an additional 652 (9% increase) identified proteins compared to 24 h (Supplementary Table 3).

Of the DAPs identified in live pathobiont treatment at 24 h, downregulated immune markers included FOSL2 (AP-1 subunit) and CASP4;CASP5 (pyroptosis initiators) (Supplementary Fig. 2A). CARD19 (NF-κB suppressor) was also downregulated, while MIF, IL18 (inflammatory cytokines), GRN (progranulin), and FTL (ferritin siderophore subunit) were upregulated. Despite these markers, GO analyses showed no hits for immunological pathways (Supplementary Fig. 2B, C). No features passed the threshold for enrichment for KEGG and Reactome pathway analyses.

In the 3 h experiment, flagellin treatment produced the most DAPs, and with a different range of immune-related markers to live pathobionts at 24 h (Supplementary Fig. 2D–F). CCL20 and CXCL5 (inflammatory chemokines), ICAM1 (leukocyte adhesion molecule), JUNB (AP-1 subunit), BCL3 (NF-κB regulator), TNFAIP3 (NF-κB activity suppressor) and TNIP1 (interacts with TNFAIP3) were upregulated, while NFKBIE (NF-κB inhibitor) was downregulated (Supplementary Fig. 2D). Upregulated proteins were enriched in T cell activation and TNF/IL17 signalling pathways (Supplementary Fig. 2E, F). Except for NFKBIE and TNIP1, the same markers were differentially abundant in flagellin + LPS treatment, with similar inflammatory GO and KEGG pathway enrichment (Supplementary Fig. 2G–I). For the heat-killed pathobiont treatment, no immune markers were identified in the DAPs from the 3 h experiment. Furthermore, no features passed the threshold for enrichment for GO, KEGG, or Reactome enrichment analyses.

### A combination of basolateral flagellin and apical LPS increased eight inflammatory cytokines and enhanced proteomic differentiation between treated and untreated PIOs

With both the live and heat-killed pathobiont cocktails failing to induce IL8 secretion, we focused on the use of flagellin and LPS. Furthermore, due to poorer proteomic differentiation between conditions at 24 h, we continued with shorter incubation periods with these stimuli. Although initially guided by previous studies using purified bacterial components like LPS^28,31,37^, we were also concerned that micromolar concentrations of flagellin and LPS were excessively high, potentially impacting the biology of PIO responses. For high LPS, this could mean excessive increases in apoptosis and mucosal damage^38^. For high flagellin, it could cause a saturation of the dose-response curve during interactions with TLR5, that may lead to a reduction in the response induced^39,40^. Therefore, we next trialled lower concentrations that had also been used in the literature^29,41^. Additionally, since flagellin-detecting TLR5 is thought to be primarily localised on the basolateral surface of the gut epithelium^42^, we hypothesized that adding flagellin basolaterally might induce a more robust response and generate greater changes within the PIO proteomes, while also facilitating the use of a lower concentration.

**Fig. 2:**
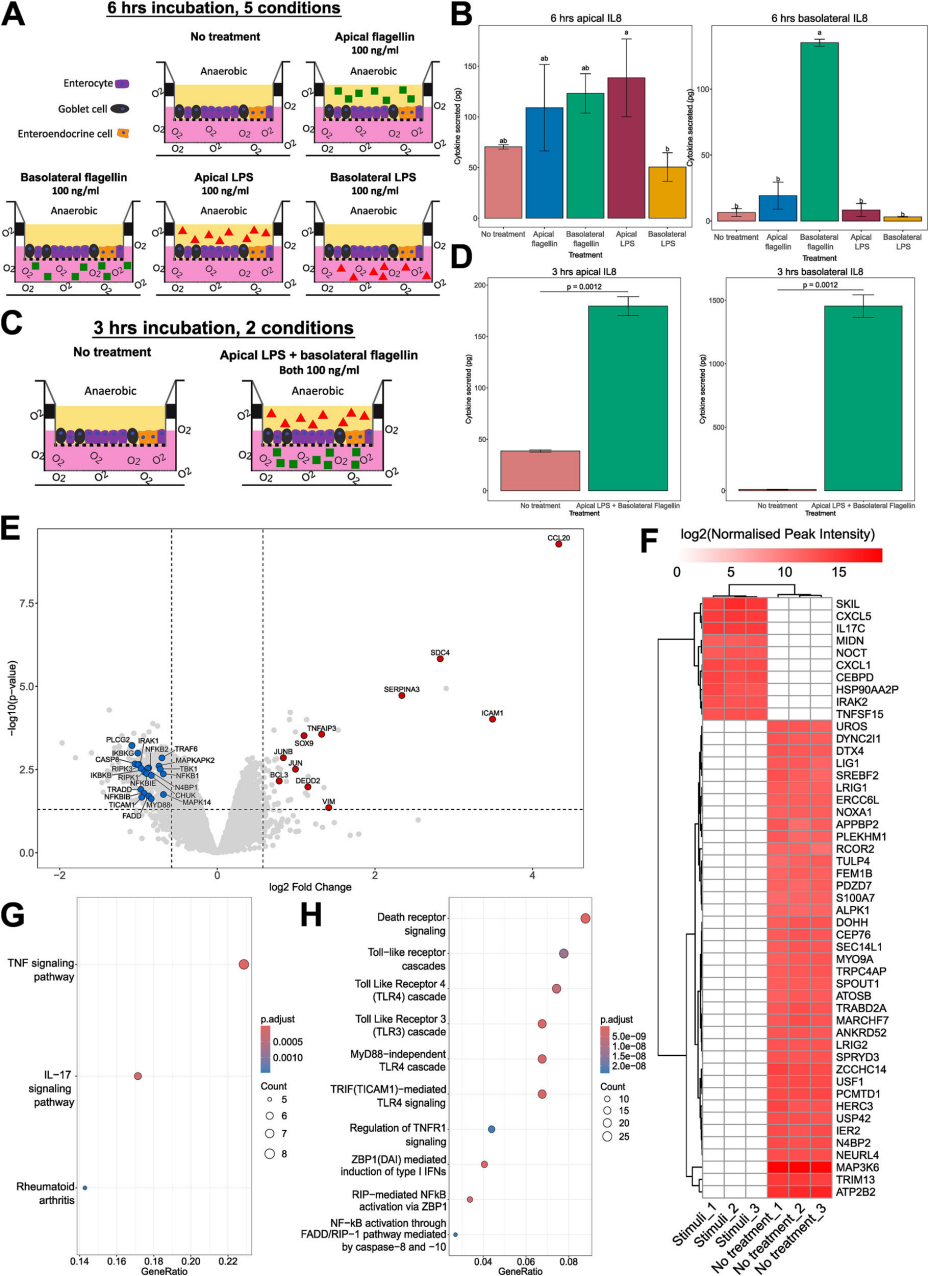
Comparisons of the effects of incubating preterm infant-derived intestinal epithelial organoids (PIOs) with either flagellin (100 ng/ml) or lipopolysaccharide (LPS) (100 ng/ml), added either apically or basolaterally, for 6 h, and a 3 h incubation with basolateral flagellin and apical LPS (both 100 ng/ml). **A** Schematic of PIO 6 h inflammation assay. **B** Bar plots showing the changes in both apical and basolateral IL8 secretion from PIOs, following 6 h incubation. Within each plot, conditions with the same letters are not significantly different. **C** Schematic of PIO 3 h inflammation assay. **D** Bar plots showing the changes in both apical and basolateral IL8 secretion from PIOs, following 3 h incubation. **E** Volcano plot showing changes in the proteomes of PIOs following 3 h incubation with apical LPS and basolateral flagellin versus untreated PIOs. The thresholds used for differential abundance are marked with dashed lines (adjusted *p* ≤ 0.05 and log2(foldchange) ≥ +/− 0.58). Differentially abundant proteins of interest are labelled on the plot, with blue signifying downregulation, and red upregulation. **F** Heatmap showing abundances of proteins from the 3 h incubation experiment, excluded from Limma fold change analysis due to being undetected in one or other condition. **G** KEGG pathways that were significantly enriched within PIO proteins upregulated during treatment with inflammatory stimuli for 3 h. **H** Reactome pathways that were significantly enriched within PIO proteins downregulated during treatment with inflammatory stimuli for 3 h.

We first trialled a 6 h incubation with either flagellin or LPS stimuli at 100 ng/ml (1000× lower than previous), added apically or basolaterally (Fig. 2A). Apical and basolateral flagellin, as well as apical LPS, induced higher average levels of apical IL8 secretion than the no treatment condition. However, none of these increases were statistically different from no treatment by Tukey’s test (all *p* > 0.05; Fig. 2B). Indeed, the only statistically significant observation was for greater apical secretion of IL8 induced by apical LPS compared to basolateral LPS (*p* = 0.02), signifying apical application of LPS to be most optimal (Fig. 2B). Conversely, only basolateral flagellin triggered a large magnitude and significant increase in basolateral IL8 secretion compared to no treatment (*p* < 0.001), signifying basolateral application of this flagellin to be optimal (Fig. 2B).

Based on these results, we next trialled a combination of apical LPS, with basolateral flagellin, both at the lower concentration of 100 ng/ml (Fig. 2C). This experiment was run for 3 h, following the improvement in proteomics output described above for this incubation time. We also quantified a wider range of cytokines, based on those detected in 3 h and 24 h proteomics data. This stimuli combination induced a significant increase in both apical and basolateral IL8 secretion compared to no treatment (both *p* = 0.0012; Fig. 2D). There were also significant increases in apical secretion of TNFα, CCL2 and CCL7 (all *p* < 0.05; Supplementary Fig. 3), and basolateral secretion of TNFα, CCL2, CCL7, CXCL5 and CCL20 (all *p* < 0.05; Supplementary Fig. 4).

Proteomics identified more proteins in this experiment than the previous two (Supplementary Table 3) and differential abundance analysis found 471 DAPs (53 upregulated, 418 downregulated) which also represents an increase on previous flagellin and LPS trials. In the presence of inflammatory stimuli, immune activation markers were upregulated (CCL20, ICAM1, SERPINA3, TNFAIP3, JUN, JUNB, BCL3, SDC4), as well as markers of tissue remodelling (SDC4, SOX9, DEDD2, VIM, SERPINA3) (Fig. 2E). Among the downregulated proteins was a group of 21 that are all part of either TLR or TNFα signalling cascades (Fig. 2E). Proteins that were detected in all replicates of one condition but not the other, and thus were excluded from fold change analysis, were also identified (Fig. 2F). Multiple markers of immune activation were uniquely present in the stimuli condition (CXCL5, IL17C, CXCL1, CEBPD, IRAK2, TNFSF15), while an additional pathogen-associated molecular pattern (PAMP) sensor and immune activator (ALPK1) was uniquely abundant within no treatment PIOs. Lists of DAPs and unique proteins were combined and pathway analyses conducted. KEGG pathways for TNF and IL17 signalling were highly enriched within the upregulated proteins (Fig. 2G), while responses to LPS and chemokines, as well as regulation of extrinsic apoptosis, were also enriched among GO terms (Supplementary Fig. 5A). Downregulated proteins were significantly enriched for multiple innate immune Reactome pathways relating to NF-κB and TLRs (Fig. 2H), with similar hits also found for GO terms and KEGG pathways (Supplementary Fig. 5B, C). As a guide for other researchers, a summary of all cytokines detected within our PIO model during all of the inflammation induction experiments is shown in Table 1.

**Table 1:** Summary of all cytokines and chemokines detected during all inflammation induction experiments.

Cytokine or chemokine	Detected in
CCL2	Cytokine assay
CCL7	Cytokine assay
CCL20	Cytokine assay/Proteomics
CXCL1	Proteomics
CXCL5	Cytokine assay/Proteomics
IL1RA	Cytokine assay
IL8	Cytokine assay
IL17C	Proteomics
IL18	Cytokine assay/Proteomics
MIF	Cytokine assay/Proteomics
TNFα	Cytokine assay

## Discussion

The current study sought to establish effective stimuli for reproducibly inducing an inflammation phenotype in PIOs, paving the way for subsequent experimental research testing novel strategies to reduce inflammation and improve health. By systematically testing different stimuli, including live and dead pathobiont consortia and defined microbial components (flagellin and LPS), as well as different co-culture durations, we provide important data to facilitate study design.

Our findings demonstrate that purified stimuli, i.e. flagellin and LPS, elicit a markedly different immune response from PIOs compared with live or dead pathobionts. The response to purified stimuli was characterised by raised chemokine production, with increases in IL8 secretion, and CCL20 and CXCL5 abundances^43^. These markers were absent from the response to live pathobionts, which instead triggered increases in different pro-inflammatory cytokines, namely IL18 and MIF^44^. Upregulation of a subunit of the siderophore ferritin also indicated a possible competition for iron with the live bacteria^45^, an aspect of host-microbe interactions that would be absent when using purified stimuli or dead pathobionts. Heat-killed pathobionts appeared to be the least effective at promoting inflammation, failing to induce either IL8 secretion or the upregulation of any immune related proteins. Overall, our results demonstrate that distinct inflammatory pathways appear to be engaged in PIOs depending on the nature of the stimulus used.

When using live pathobionts, a longer incubation period was required to observe significant proteomic alterations. This may have been due to the need for PAMPs to be extracted from the bacterial cells by scavenger receptors, which promote PAMP delivery to immune sensors such as TLRs^46^. This is thought to be a rate-limiting step in immune activation when using whole bacteria for stimulation rather than purified ligands. In contrast, the near absence of DAPs in the 24 hr proteomics dataset for LPS and flagellin treatments, despite increases in IL8 secretion, likely reflects a transient immune response, wherein the inflammatory cascade is mounted rapidly but subsequently downregulated, allowing PIOs to return to baseline by this time point. This suggests the need for shorter incubation times when using purified stimuli, to capture early immune dynamics.

The final model tested, in which LPS was applied apically and flagellin basolaterally, induced a robust pro-inflammatory cytokine secretion profile and the widest changes in the proteome, constituting the strongest response obtained from PIOs during these experiments. It is important to note that this response was induced using 1000× lower concentrations of the stimuli (both 100 ng/ml) than were first employed. This highlights the need to consider how using biologically extreme quantities of stimuli may impact experimental models.

A key observation from the proteomics was the downregulation of signalling cascade components that are downstream of TLR4 and TLR5^46^, with suppression MyD88, IRAK1, TRAF6, TICAM1, NFKB1 and NFKB2. These last two proteins are particularly of interest, as they are precursors for the NF-κB subunits p50 and p52, respectively^47^. They are two of the five proteins that form NF-κB homo- or heterodimeric transcription factors which promote expression of pro-inflammatory genes. Prolonged LPS stimulation has been shown to downregulate TLR4 and the MyD88 pathway^48^, promoting LPS tolerance, which may be the mechanism underpinning this observation. BCL3 upregulation could also cause suppression of NF-κB-mediated inflammation, as it has been shown to inhibit NF-κB target gene expression in certain contexts^49^. Negative feedback regulation was also evident with TNFAIP3 (A20) upregulation, which inhibits pro-inflammatory signalling by inactivating TRAF6^50,51^. The process of LPS toleration has been shown to induce TNFAIP3 upregulation^52,53^. Additionally, the observed increase in TNFα secretion will likely have contributed to TNFAIP3 upregulation^54^. The TNFα signalling pathway was enriched within the upregulated proteins, suggesting this cytokine was indeed acting on cells within the organoids, in either an autocrine or paracrine manner^55^. Negative feedback of immunity by TNFAIP3 constitutes a homeostatic system which prevents an uncontrolled pro-inflammatory response and tissue damage, suggesting the model successfully achieved inflammation of the PIOs during incubation.

The AP-1 transcription factor subunits JUN and JUNB^46^ were upregulated, suggesting heightened and continued AP-1-dependent transcriptional activity. This coincided with reduced NF-κB transcription factor abundance, pointing to a shift away from the NF-κB pathway and towards MAPK-mediated inflammation. Differences in the relative activities of these two pathways have been observed previously, with NF-κB subsiding while MAPK signalling surges^56,57^. Further, the two pathways have differing thresholds for activity, with NF-κB acting as a general low level bacterial sensor and MAPK serving as an alarm for increasing threat levels^57^. CEBPD, a transcription factor known to be induced by LPS^58^, was also only detected in the presence of stimuli. This protein regulates inflammatory genes^59^, in particular enhancing NF-κB mediated transcription in response to persistent TLR4 stimuli^60^. It may therefore have played an important role in initiating inflammation within our model. Secretion of multiple pro-inflammatory cytokines, namely IL-8, TNFα, CXCL5, CCL2, and CCL7^44^, was also raised, as well as protein abundances of IL17C and CXCL1. Furthermore, many of these cytokines are chemokines (CCL20, IL8, CXCL1, CXCL5, CCL2, and CCL7)^43^, indicating activation of sustained immune cell recruitment. The upregulation of ICAM1 would further facilitate this recruitment, as an intercellular adhesion molecule that binds to leukocytes at the site of inflammation^61,62^.

Increased IL-17C is of particular importance, as it is known to be produced by intestinal epithelial cells in response to bacterial pathogens and functions in an autocrine manner to amplify innate host defence^63^. Enrichment of the IL17 signalling pathway was found in the upregulated proteins, suggesting this cytokine was directly acting on the organoids. Syndecan-4 (SDC4) is also a marker of inflammation, as LPS, flagellin and TNFα have been shown to induce its expression via NF-κB, and its upregulation also shows that NF-κB transcription factors were active despite their downregulation^64–66^. This adhesion receptor for tissue damage does however limit inflammation and injury^65,67^, indicating again that homeostatic mechanisms to control inflammation were active in the organoids. All of these observations support the notion that despite MyD88 pathway suppression, inflammation induction continued via AP-1 transcription and cytokine signalling.

In addition to inflammation, PIOs exhibited signs of tissue regeneration, remodelling and wound healing. The transcription factor SOX9^68^ plays a critical role in driving the regenerative response within the intestinal epithelium, both in vivo and in organoids^69,70^, and so its elevation in the presence of stimuli suggests an induction of this response. Additionally, there are NF-κB binding sites within the *Sox9* promoter region^71^, therefore the increased SOX9 abundance gives further evidence that NF-κB transcription was active in the stimulated organoids. Furthermore, upregulation of the cytoskeleton filament protein vimentin (VIM) is seen as a marker of cells undergoing the epithelial-mesenchymal transition, which would facilitate tissue remodelling and wound healing^72–74^. Finally, SERPINA3 inhibits proteases that mediate inflammation, particularly those that degrade the extracellular matrix^75^. Its upregulation in the stimulated organoids could thus exert a protective effect by helping to resolve inflammation and promote wound healing.

Apoptosis, a form of programmed cell death, is also important for tissue remodelling, regeneration and wound healing^76^. This process can be initiated by a ligand, such as TNFα, binding to TNF receptor I, which then recruits TRADD^44,77^. This receptor-TRADD complex is then internalised, whereupon it is bound by FADD, which in turn recruits CASP8, activating it and triggering apoptosis. TRADD, FADD and CASP8 were all downregulated within stimulated organoids, indicating an inhibition of this apoptotic pathway. This may be a protective response to the increase in TNFα secretion from stimulated PIOs to make cells less sensitive to this ligand and prevent an excessive induction of apoptosis. An alternate apoptotic pathway was however upregulated, in the form of DEDD2^78,79^. This protein is targeted to the nucleolus of cells and its upregulation is sufficient to induce apoptosis^80^. Overall, PIOs may be rebalancing tissue regeneration and apoptosis in the face of sustained inflammatory pressure.

A limitation of this study is that individual bacterial strains were not systematically tested within the organoid system. Specific pathobionts may be more immunogenic than others and thus could have induced a stronger inflammatory response. Notwithstanding, the pathobiont cocktail allows for a more global assessment of whole community impacts and further work could systematically explore individual bacterial strains from the patient population of interest.

A further limitation is that microbe-microbe interactions and changes to bacterial viability within the live pathobiont cocktail during co-culture were not assessed. The possibility therefore remains that community dynamics may have differed between replicates, which could have introduced additional heterogeneity into the system. It also remains unknown whether specific species dominated the community after incubation and could thus have the observed induced effects attributed to their activities. Investigating community composition during co-culture would thus be a useful direction for future applications of this pathobiont cocktail model.

Taken together, these findings highlight that treatment of PIOs with a combination of apical LPS and basolateral flagellin for 3 h induced a robust pro-inflammatory immune response, featuring a complex interplay between inflammation, immune tolerance, and tissue remodelling. Use of either live or dead bacteria failed to induce similarly detectable or complete responses. We therefore identified this ‘LPS + flagellin’ model as the most robust and reproducible approach to studying inflammation in the preterm intestinal epithelium. We hope that our characterisation of this model can serve as a reference for validation of future organoid experiments utilising this or similar methods. Establishment of a baseline inflammatory response in this organoid system provides a benchmark for mechanistic investigations, improving reproducibility between such experiments and enabling assessment of organoid health and detection of deviations that may confound experimental interpretations. Ultimately, such a model will facilitate future research into therapeutic modulation and disease prevention strategies.

## Methods

### Ethics and sample collection

Preterm infants under 32 weeks gestation were born or transferred to a single tertiary level Neonatal Intensive Care Unit in Newcastle upon Tyne, UK, and participated in the Supporting Enhanced Research in Vulnerable Infants (SERVIS) study after written informed parental consent, as approved by the National Research Ethics Service Committee North East and North Tyneside 2 10/H0908/39, of Newcastle Hospitals NHS Foundation Trust. Intestinal tissue samples used to generate organoid cell lines were salvaged following surgical resection. Stool samples were regularly collected from nappies/diapers of preterm infants into sterile collection pots by nursing staff. Samples were initially stored at −20 °C before being transferred to −80 °C for long term storage.

### Bacterial pathobiont isolation and identification from preterm stool samples

Stool samples were thawed on ice and initially diluted roughly 1:10 w/v in sterile anaerobic phosphate buffered saline (PBS). 10-fold serial dilutions were then performed using sterile anaerobic PBS and various dilutions (typically 10-2 and 10-4) were cultured by adding 100 μl of inoculum onto an agar plate, before spreading to cover the surface of the agar plate and incubating for up to 96 h. Numerous different agar media were used including brain heart infusion (BHI), Transoligosaccharide propionate (TOS), Bifidus Selective Medium (BSM), De Man, Rogosa and Sharpe (MRS), fastidious anaerobe agar (FAA), and yeast extract-peptone-dextrose (YPD). Unique appearing colonies based on morphology, colour, and size were sub-cultured twice. Full-length 16S rRNA gene sequencing (27F 5′-AGAGTTTGATCCTGGCTCAG3’; 1492R 5′-GGTTACCTTGTTACGACTT-3′) and matrix-assisted laser desorption ionization-time of flight mass spectrometry (Bruker MALDI-TOF MS) of single fresh colonies were used to initially identify isolates to genus or species level. Isolates were added to glycerol for long term storage at −80 °C.

### Organoid media production

Organoid media were made as previously described by Stewart et al.^6^.

Complete Media Growth Factor negative (CMGF−): 500 ml Advanced DMEM/F12, 5 ml GlutaMAX 100×, 5 ml HEPES 1 M.

Complete Media Growth Factor positive (CMGF+) in a volume of 500 ml: 78 ml CMGF−, 250 ml Wnt3A-conditioned media produced from ATCC CRL-2647 cells (ATCC), 100 ml R-spondin-conditioned media produced from R-Spondin1 expressing 293T cells (Merck), 50 ml Noggin-conditioned media produced from 293-Noggin cells(24), 10 ml B27 (50×), 5 ml N2 (100×), 5 ml nicotinamide (10 mM), 1 ml N Acetylcysteine (1 mM), 500 μl Gastrin (10 nM), 500 μl A83 (500 nM), 166 μl SB202190 (10 μM), 50 μl EGF (50 ng/ml).

High Wnt medium in a volume of 500 ml: 250 ml CMGF+, 250 ml Wnt3A-conditioned media.

Differentiation (DIF) medium in a volume of 500 ml: 458 ml CMGF−, 25 ml Noggin conditioned media, 10 ml B27 (50×), 5 ml N2 (100×), 1 ml N Acetylcysteine (500 mM), 500 μl Gastrin (100 μM), 500 μl A83 (500 μM), 50 μl EGF (500 μg/ml).

### Establishment and culture of preterm infant intestinal-derived organoids

Intestinal epithelial organoid lines were established from intestinal crypts isolated from preterm neonate small intestine tissue, as described by Stewart et al.^6^. Briefly, tissue was minced, washed with chelating solution, antifungals and antibiotics, and crypt cells extracted by gentle shaking in EDTA. Extracted cells were then suspended within phenol-red free and growth factor reduced Matrigel basement membrane matrix (Corning), which was then pipetted as small dots into the wells of a 24 well plate, at a maximum of three dots per well. The Matrigel was set by incubating at 37 °C for 30 min. Following polymerization of the Matrigel, 500 μl High Wnt growth medium was added to each well and the organoids left to grow from the crypt cells, with incubation at 37 °C and 5% CO_2_. Media was changed for fresh every Monday, Wednesday and Friday. Organoids were passaged by removing spent medium and adding 300 μl 0.05% Trypsin-EDTA (Gibco) per well. The trypsin was then pipetted up and down to break up the Matrigel and suspend the organoids. Organoids were incubated in trypsin at 37 °C and 5% CO_2_ for 5 min, with 350 μl fetal bovine serum (Merck) then added to stop trypsinisation. Following centrifugation at 363 G for 5 min, supernatant was removed, and the organoids resuspended in ice cold Matrigel, the volume of which depended on the number of wells being seeded. The Matrigel was then pipetted as small dots into the wells of a 24 well plate, at a maximum of three dots per well and set by incubating at 37 °C for 30 min. Following polymerization of the Matrigel, 500 μl High Wnt growth medium was added to each well and the organoids left to grow, with incubation at 37 °C and 5% CO_2_. All organoid experiments were conducted with cell line NCL 27 within passages 10–15. This line was established from a patient born at 24 weeks gestation, using ileum tissue salvaged from surgery performed on day of life 10 due to the development of necrotising enterocolitis.

### Organoid monolayer co-culture with flagellin, lipopolysaccharide, live pathobionts and heat-killed pathobionts

Three dimensional organoids were seeded onto two dimensional monolayers in 6.5 mm Transwells (Corning #3470), following the protocol described by Fofanova et al.^23^. Briefly, 3D organoid cultures were processed into single cell suspensions by washing with EDTA, trypsinisation (0.05% trypsin-EDTA) and passage through a 40 μm nylon cell strainer (Corning). Cells were then resuspended in CMGF+ growth medium (1.8 × 10^6^ cells per ml) and seeded onto Transwells, pre-coated with diluted (1:40 in cold PBS) Matrigel. The basolateral medium volume used was 650 μl and the apical medium volume in the Transwell was 200 μl. Growth medium was replaced with DIF medium after 2–3 days, once trans-epithelial electrical resistances (TEERs) were near 300 Ω, indicating monolayer confluence. Monolayers were differentiated for four days prior to experiments. Monolayers were then placed into the anaerobic co-culture system, which was in turn placed into an anaerobic chamber. Oxygen is flowed through the base of the co-culture unit, maintaining aerobic conditions on the basolateral sides of the monolayers, while the apical sides remain under the anaerobic conditions of the chamber. Thus, the physiological oxygen gradient of the gut epithelium is recreated in this model. The inflammatory stimuli used were lipopolysaccharide (LPS) from *Escherichia coli* 0111:B4 (InvivoGen) and flagellin from *Salmonella typhimurium* (InvivoGen), concentrated stocks of which were made using DIF medium. For the cocktail of live pathobionts, nine preterm infant isolates were used (Supplementary Table 1). Glycerol stocks of each were streaked out on BHI agar and incubated overnight. Single colonies were then picked and inoculated in 5 ml BHI broth and grown for 2 days. A day prior to the experiment, 250 μl of these starter cultures were then inoculated into a fresh 5 ml of BHI, constituting a 5% inoculum, and incubated for 24 h. The OD600 of these cultures was measured and the volume needed to give OD 1 in 1 mL DIF medium was calculated. These volumes were then centrifuged at 5000 × *g* for 5 min, and the pellets resuspended in DIF medium. All nine strains were then pooled in equal volume, having been enumerated separately (Supplementary Table 1). The total OD of this suspension was therefore 1. Accordingly, the total number of bacteria added to the apical side of organoid monolayers in 200 μl of medium was 8.68 × 10^7^ cells. For heat-killed pathobionts, 5 ml cultures of the same nine strains in BHI broth were prepared as above and incubated for 72 h. The OD600 of each culture was measured and volumes calculated to resuspend each to OD 3. Equal volumes of each were pooled into a sterile conical flask, which was then autoclaved at 121 °C for 15 min. To confirm the absence of live bacteria, 100 μl of autoclaved culture was streaked out on a BHI plate and incubated in the anaerobic chamber for 5 days. The OD600 of the autoclaved culture was measured again and the volume required to give OD 2 in 1 ml DIF medium was calculated. This volume was centrifuged at 5000 × *g* for 5 min and the pellet resuspended in 1 ml DIF. Prior to the experiment, DIF medium to be used on the apical side of the monolayers was placed in the anaerobic chamber overnight to remove any oxygen. For the experiment, the following conditions were set up in triplicate across the monolayers: 1) No stimuli (‘No treatment’), 2) + apical flagellin (100 μg/ml), 3) + apical LPS (100 μg/ml), 4) + live pathobionts (OD 1), 5) + heat-killed pathobionts (OD 2). After completion of anaerobic co-culture set up within the anaerobic chamber, the organoid monolayers were incubated within the system for 1 h, to allow equilibration to the anaerobic conditions. Treatments were then added following this incubation. Two separate experiments with these treatment conditions were performed; one with a 24 h incubation and the other with 3 h. Following incubation, apical and basolateral supernatants were harvested for cytokine assays. Organoid monolayers were harvested for proteomics. The base of each Transwell was excised using a sterile scalpel and placed into 300 μl ice cold PBS, kept on ice. The PBS was pipetted up and down repeatedly to dislodge the monolayer from the Transwell base and the base was removed from the PBS. All samples were then centrifuged at 363 × *g*, at 4 °C for 10 min and the supernatant removed. The dry cell pellets were then stored at −80 °C, until needed for proteomics sample preparation. These and subsequent organoid experiments were performed once, with three separate organoid monolayers used per treatment, facilitating three replicates of each treatment within each experiment.

### Organoid monolayer co-culture with apical or basolateral flagellin and LPS, at lower concentrations

Monolayers were established as described above. The same flagellin and LPS inflammatory stimuli were used as above. For the experiment, the following conditions were set up in triplicate across the monolayers: 1) No stimuli (‘No treatment’), 2) + apical flagellin (100 ng/ml), 3) + basolateral flagellin (100 ng/ml), 4) + apical LPS (100 ng/ml), 5) + basolateral LPS (100 ng/ml). After completion of anaerobic co-culture set up within the anaerobic chamber, the organoid monolayers were incubated within the system for 1 h, to allow equilibration to the anaerobic conditions. Treatments were then added following this incubation. This experiment was run for 6 h. Following incubation, apical and basolateral supernatants were harvested for cytokine assays.

### Organoid monolayer co-culture with apical LPS and basolateral flagellin

Monolayers were established as described above. The same flagellin and LPS inflammatory stimuli were used as above. For the experiment, the following conditions were set up in triplicate across the monolayers: 1) No stimuli (‘No treatment’), 2) + apical LPS + basolateral flagellin (both 100 ng/ml). After completion of anaerobic co-culture set up within the anaerobic chamber, the organoid monolayers were incubated within the system for 1 h, to allow equilibration to the anaerobic conditions. Treatments were then added following this incubation. This experiment was run for 3 h. Following incubation, apical and basolateral supernatants were harvested for cytokine assays. Monolayers were also harvested for proteomics, as described above.

### Cytokine assays

Secreted interleukin-8 (IL-8) was measured using a DuoSet ELISA kit, following the manufacturer’s protocol. All other cytokines were measured using custom U-Plex assays (Meso Scale Discovery Inc.), following the manufacturer’s protocol. Outliers were identified by calculating robust/modified z scores for each data point. This method uses the median to calculate median absolute deviation. Any data point with a robust z score greater than +3.5 or less than −3.5 was removed from the dataset. For experiments with more than two conditions, ANOVA followed by a Tukey’s HSD test were used to perform statistical analysis of cytokine secretion data. When only two conditions were set up, an unpaired *t* test was used. *P* < 0.05 was set as the threshold for statistical significance.

### Proteomics sample preparation, mass spectrometry and data analysis

Pelleted organoid monolayers were lysed by addition of 50 mM triethyl ammonium bicarbonate (TEAB) pH 8.5 with 5% (w/v) SDS and 1× cOmplete protease inhibitor cocktail (Roche). Samples were sonicated using a UP200St ultrasonic processor (Hielscher) at 90 W, 45 s pulse, 15 s rest, three times. Protein concentration was quantified using Pierce BCA Protein Assay (ThermoFisher Scientific). Samples (10 µg) were then denatured with 5 mM tris(2-carboxyethyl)phosphine (TCEP) at 60 °C for 15 min, alkylated with 30 mM iodoacetamide at room temperature (20 °C) for 30 min in the dark, and acidified to a final concentration of 2.5% phosphoric acid. Samples were then diluted eightfold with 90% MeOH 10% TEAB (pH 7.2) and added to the S-trap (Protifi)^81,82^ micro columns. The manufacturer-provided protocol was then followed, with a total of five washes in 90% MeOH 10% TEAB (pH 7.2), and trypsin in 50 mM TEAB added at a ratio of 1:10 enzyme:protein and digestion performed for 18 h at 37 °C or 3 h at 47 °C. Peptides were dried using a vacuum concentrator and stored at −80 °C, and immediately before mass spectrometry were resuspended in 0.1% formic acid (both 3 h incubation experiments) or 2% acetonitrile 0.1% trifluoracetic acid (24 h incubation experiment).

For the 3 h incubation experiments, liquid chromatography (LC) was performed using an Evosep One system with a 15 cm Aurora Elite C18 column with integrated Captivespray emitter (IonOpticks), at 50 °C. Buffer A was 0.1% formic acid in HPLC water, buffer B was 0.1% formic acid in acetonitrile. Immediately prior to LC-MS, peptides were resuspended in buffer A and a volume of peptides equivalent to 500 ng was loaded onto the LC system-specific C18 EvoTips, according to manufacturer instructions, and subjected to the predefined WhisperZoom 20 SPD protocol (where the gradient is 0–35% buffer B, 200 nl/min, for 58 min, 20 samples per day are permitted using this method). The Evosep One was used in line with a timsToF-HT mass spectrometer (Bruker), operated in diaPASEF mode. Mass and IM ranges were 300–1200 *m/z* and 0.6–1.45 1/*K*_*0*_, diaPASEF was performed using variable width IM-*m/z* windows, as described previously^83^ TIMS ramp and accumulation times were 66 ms, total cycle time was ~1.2 s. Collision energy was applied in a linear fashion, where ion mobility = 0.6–1.6 1/K_0_, and collision energy = 20–59 eV.

For the 24 h incubation experiment, each sample was independently analysed on an Orbitrap Q Exactive HF mass spectrometer (Thermo Fisher Scientific), connected to an UltiMate 3000 RSLCnano System (Thermo Fisher Scientific). Peptides (1 µg) were injected on a PepMap 100 C18 LC trap column (300 μm ID × 5 mm, 5 μm, 100 Å) followed by separation on an EASY-Spray nanoLC C18 column (75 μm ID × 50 cm, 2 μm, 100 Å) at a flow rate of 250 nl min^−1^. Solvent A was water containing 0.1% formic acid, and solvent B was 80% acetonitrile containing 0.1% formic acid. The gradient used for analysis of proteome samples was as follows: solvent B was maintained at 3% for 5 min, followed by an increase from 3 to 35% B in 120 min, 35–90% B in 0.5 min, maintained at 90% B for 4 min, followed by a decrease to 3% in 0.5 min and equilibration at 3% for 10 min.

The Orbitrap Q Exactive HF was operated in positive-ion data-independent acquisition (DIA) mode. The precursor ion scan (full scan) was performed in the Orbitrap in the range of *m/z* 300–1650 with a resolution of 120,000, with an automatic gain control (AGC) target of 1 × 10^6^ at a maximum injection time of 60 ms. Subsequently, DIA scans were collected using 16 *m/z* staggered windows, with loop count set to 45, Orbitrap resolution of 30,000, AGC target of 1 × 10^6^, HCD collision energy set to 27, and maximum injection time of 55 ms.

Raw diaPASEF data files were searched using DIA-NN V 1.9^84^, using its in silico generated spectral library function, based on reference proteome FASTA files for *H. sapiens* (UP000001414, downloaded from UniProt on 11/05/2023) and a common contaminants list^85^. Trypsin specificity with a maximum of 1 missed cleavage was permitted per peptide, cysteine carbamidomethylation were set as a fixed modification. Peptide length and m/z was 7–30 and 300–1200, charge states 2–4 were included. Mass accuracy was fixed to 15 ppm for MS1 and MS2. Protein and peptide FDR were both set to 1%. All other settings were left as defaults.

Q-Exactive raw data were analysed using DIA-NN V 1.8.1, and searched against a SwissProt homo sapiens fasta files (containing 42,371 database entries with isoforms, downloaded on 2021/02/24) and contaminant FASTA (Frankenfield et al. 2022); searches were performed using default parameters. The enzyme specificity was set to consider fully tryptic peptides, and two missed cleavages were allowed. A protein and peptide false discovery rate (FDR) of less than 1% was employed. The Q-Exactive mass spectrometry proteomics data have been deposited to the ProteomeXchange Consortium via the PRIDE^86^ partner repository with the dataset identifier PXD067236, the timsToF data have been deposited with the dataset identifier PXD067233.

For post processing, the data was filtered to remove proteins that matched to a contaminant, or which contained less than two unique peptides. Intensity values were median normalised and log2 transformed. For experiments with more than two conditions, the median normalised data were compared by PERMANOVA, using the package Vegan (version 2.6.8)^87^ in R (version 4.3.0). Proteins that were present in at least two replicates of all conditions within an experiment were retained in the dataset. Those that did not meet this requirement were filtered out. Differential abundance analysis was then performed using Limma (version 3.56.2)^88^. The thresholds for differential abundance were set at a fold change of 1.5 and an adjusted *p* value ≤ 0.05. Proteins that were filtered out earlier, due to not being detected in at least two replicates of all experimental conditions, were manually studied for those uniquely detected in specific conditions, by creating a heatmap of the data with the package pheatmap (version 1.0.12)^89^. Pathway enrichment analyses of up- and downregulated proteins was performed using the package clusterProfiler (version 4.8.3)^90^, surveying for enrichments in Gene Ontology (GO), Kyoto Encyclopaedia of Genes and Genomes (KEGG) and Reactome pathways, with the threshold for enrichment set at *p* ≤ 0.05.

## Supplementary information

Supplementary Materials

## Data Availability

The Q-Exactive mass spectrometry proteomics data have been deposited to the ProteomeXchange Consortium via the PRIDE29 partner repository with the dataset identifier PXD067236, the timsToF data have been deposited with the dataset identifier PXD067233.
