# Supplementary material for: Optimising the induction of inflammation within preterm infant-derived intestinal epithelial organoids
Source: NPJ Gut Liver. 2026 Feb 2;3(1):5. doi: 10.1038/s44355-026-00054-2 (PMC12864041; doi:10.1038/s44355-026-00054-2)
Supplement: Supplementary file 1 — Supplementary Materials [file 44355_2026_54_MOESM1_ESM.pdf]

Supplementary Materials for

Optimising the induction of inflammation within preterm infant-derived intestinal epithelial organoids

Chapman et al.

Corresponding authors:
Dr Jonathan Chapman, jon.chapman@newcastle.ac.uk
Professor Christopher Stewart, christopher.stewart@newcastle.ac.uk

The file includes:
Supplementary Tables 1 to 3
Supplementary Figures 1 to 5

Supplementary Table 1. Preterm infant bacterial isolates included within the pathobiont cocktail used to test inflammation induction.

Strain ID	Species	CFU/ml within the pathobiont cocktail	CFU added to organoids as part of pathobiont cocktail
AM3	*Enterococcus faecalis*	7.22×10^{7}	1.44×10^{7}
AM72	*Staphylococcus epidermidis*	2.83×10^{7}	5.67×10^{6}
AM114	*Escherichia coli*	5.11×10^{7}	1.02×10^{7}
JC10	*Enterobacter cloacae*	7.15×10^{7}	1.43×10^{7}
JC14	*Enterococcus faecium*	2.04×10^{7}	4.07×10^{6}
JC15	*Staphylococcus haemolyticus*	2.15×10^{7}	4.30×10^{6}
MR1	*Klebsiella pneumoniae*	5.67×10^{7}	1.13×10^{7}
MR3	*Staphylococcus aureus*	5.74×10^{7}	1.15×10^{7}
VK66	*Klebsiella oxytoca*	5.48×10^{7}	1.10×10^{7}

*CFU – colony forming units

Supplementary Table 2. Change in the number of differentially abundant proteins (DAPs) identified under different thresholds.

Condition	Time (hrs)	DAPs with +/-1.5 FC threshold	DAPs with no FC threshold	Change in DAPs
Dead pathobiont	24	1	1	0
Flagellin	24	1	2	+1
Flagellin + LPS	24	2	4	+2
Live pathobionts	24	115	438	+323
Dead pathobionts	3	11	64	+53
Flagellin	3	40	224	+184
Flagellin + LPS	3	17	48	+31
Live pathobionts	3	30	133	+103

DAPs – differentially abundant proteins; FC – fold change; LPS – lipopolysaccharide

Supplementary Table 3. Number of proteins for which at least two peptides were detected during proteomics following inflammation induction experiments.

Experiment	No. proteins detected
24hrs + apical stimuli	7052
3hrs + apical stimuli	7704
3hrs + apical LPS + baso flagellin	8234

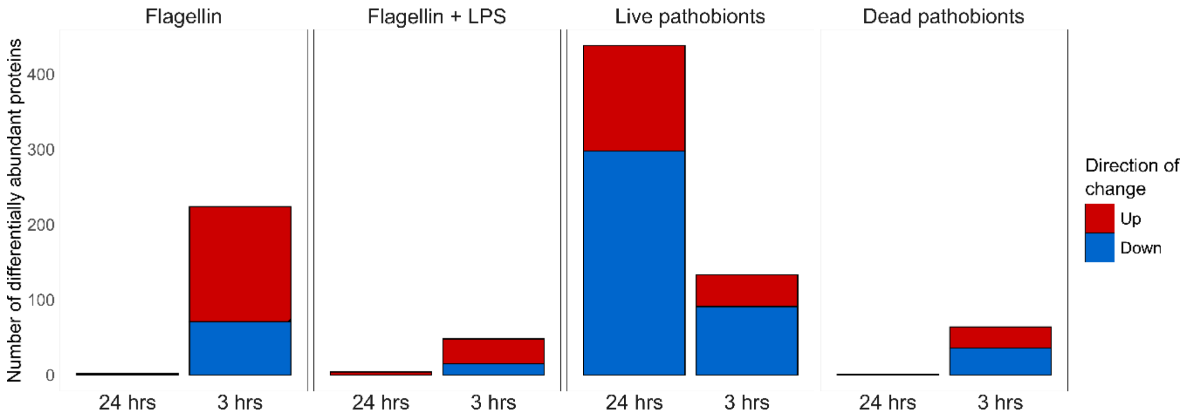

Supplementary Figure 1. Per condition comparison of the number of differentially abundant proteins identified within preterm infant-derived intestinal epithelial organoids exposed to apical lipopolysaccharide (LPS) (100 µg/ml) and flagellin (100 µg/ml), live pathobionts or heat-killed pathobionts at 24 hrs and 3 hrs incubation, with threshold for log2(foldchange) removed. The threshold for differential abundance was set at adjusted $p \leq 0.05$ only.

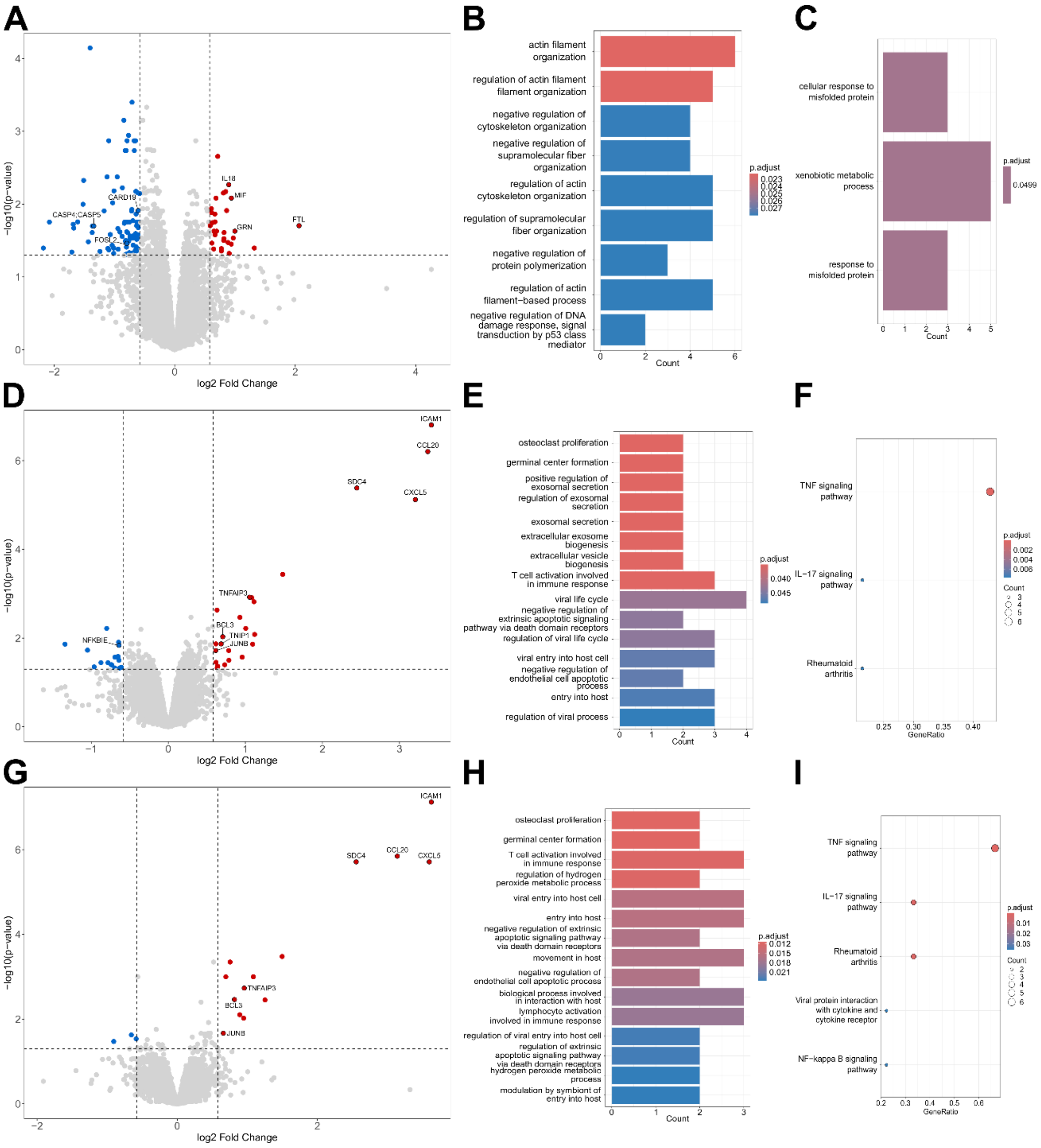

Supplementary Figure 2. Comparison of proteome changes induced within preterm infant-derived intestinal epithelial organoids (PIOs) incubated with one of: live pathobionts (24 hrs); flagellin (100 µg/ml) (3 hrs); flagellin and lipopolysaccharide (LPS) (both 100 µg/ml) (3 hrs). **(A)** Volcano plot showing changes in the proteomes of PIOs following 24 hr incubation with live pathobionts versus untreated PIOs. **(B)** Gene ontology (GO) biological process terms that were significantly enriched within PIO proteins upregulated during treatment with live pathobionts for 24 hrs. **(C)** GO biological process terms that were significantly enriched within PIO proteins downregulated during treatment with live pathobionts for 24 hrs. **(D)** Volcano plot showing changes in the proteomes of PIOs following 3 hr incubation with flagellin versus untreated PIOs. **(E)** GO biological process terms that were significantly enriched within PIO proteins upregulated during treatment with flagellin for 3 hrs. **(F)** KEGG pathways that were significantly enriched within PIO proteins upregulated during treatment with flagellin for 3 hrs. **(G)** Volcano plot showing changes in the proteomes of PIOs following 3 hr incubation with flagellin and LPS versus untreated PIOs. **(H)** GO biological process terms that were significantly enriched within PIO proteins upregulated during treatment with flagellin and LPS for 3 hrs. **(I)** KEGG pathways that were significantly enriched within PIO proteins upregulated during treatment with flagellin and LPS for 3 hrs. **(A,D and G)** The thresholds used for differential abundance are marked with dashed lines (adjusted p ≤0.05 and log2(foldchange) ≥ +/- 0.58). All upregulated proteins are shown in red, and all downregulated proteins are shown in blue. Immune-related proteins of interest are labelled.

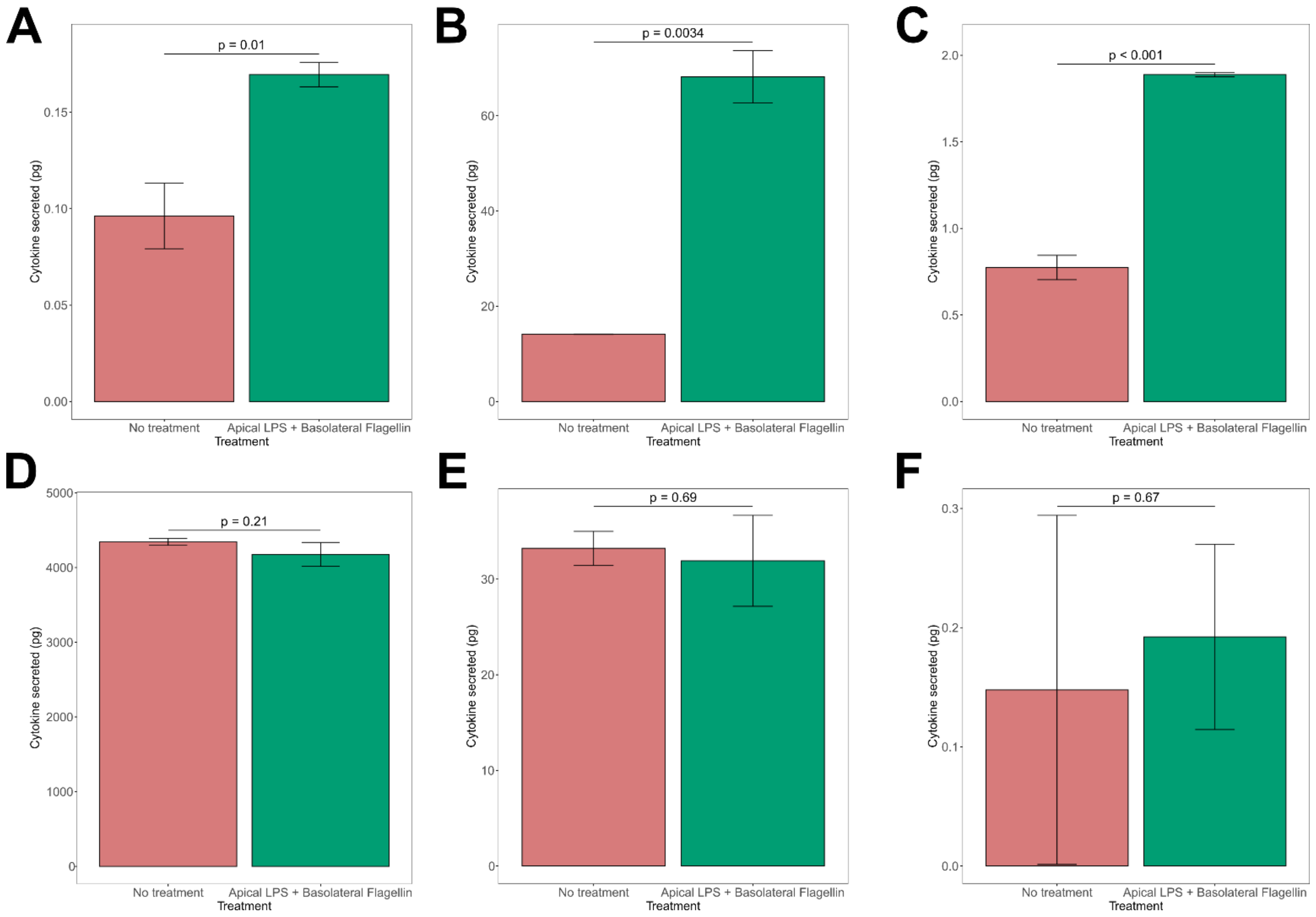

Supplementary Figure 3. Bar plots showing the changes in apical secretions of additional cytokines from preterm infant-derived intestinal epithelial organoids, following 3 hrs incubation with apical lipopolysaccharide and basolateral flagellin (both 100 ng/ml) . Assays for CXCL5 and CCL20 were not performed due to lack of apical sample. **(A)** TNFα **(B)** CCL2 **(C)** CCL7 **(D)** MIF**(E)** IL1RA **(F)** IL18

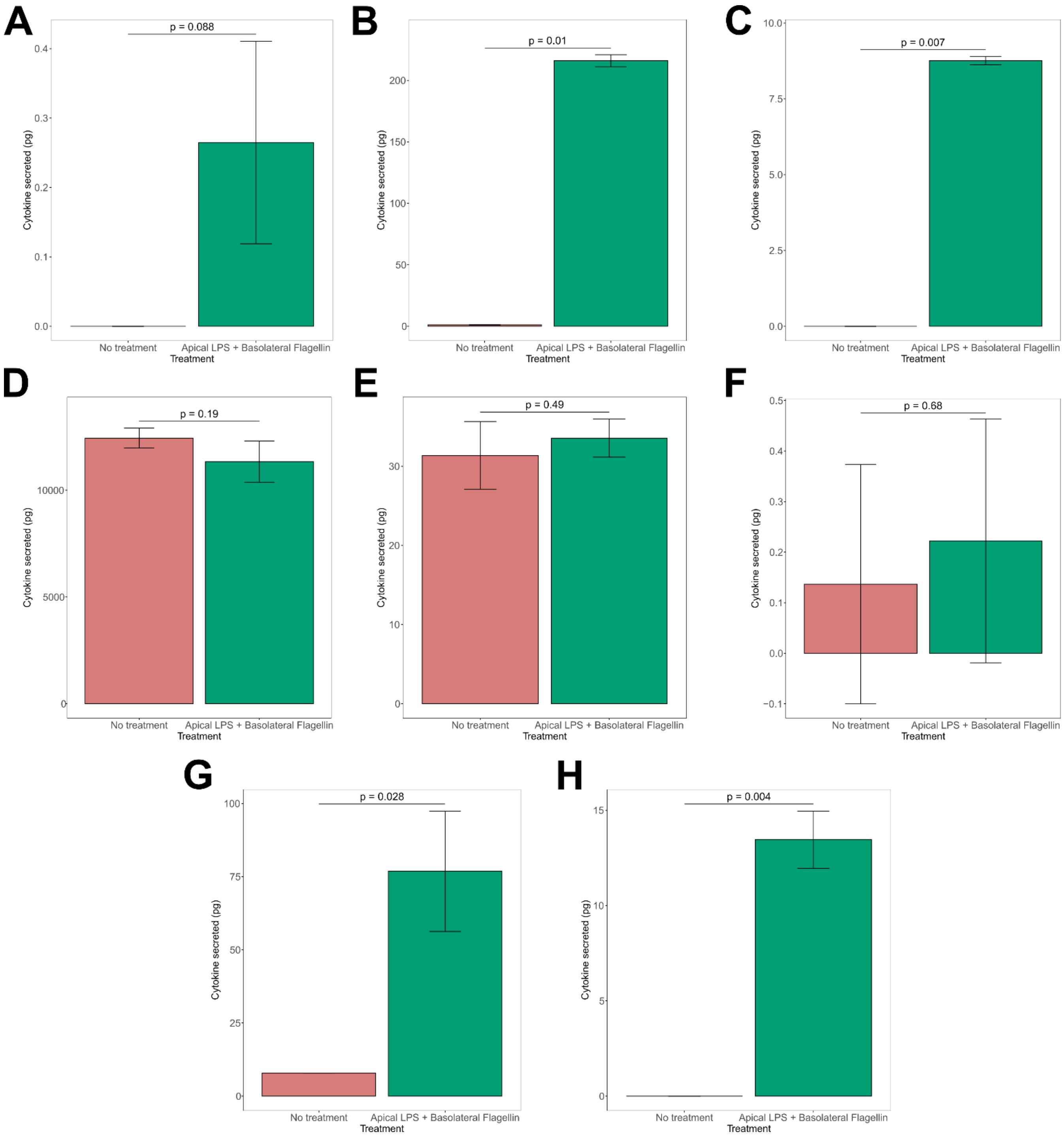

Supplementary Figure 4. Bar plots showing the changes in basolateral secretions of additional cytokines from preterm infant-derived intestinal epithelial organoids, following 3 hrs incubation with apical lipopolysaccharide and basolateral flagellin (both 100 ng/ml) . Assays for CXCL5 and CCL20 were not performed due to lack of apical sample. **(A)** TNFα **(B)** CCL2 **(C)** CCL7 **(D)** MIF **(E)** IL1RA **(F)** IL18 **(G)** CXCL5 **(H)** CCL20

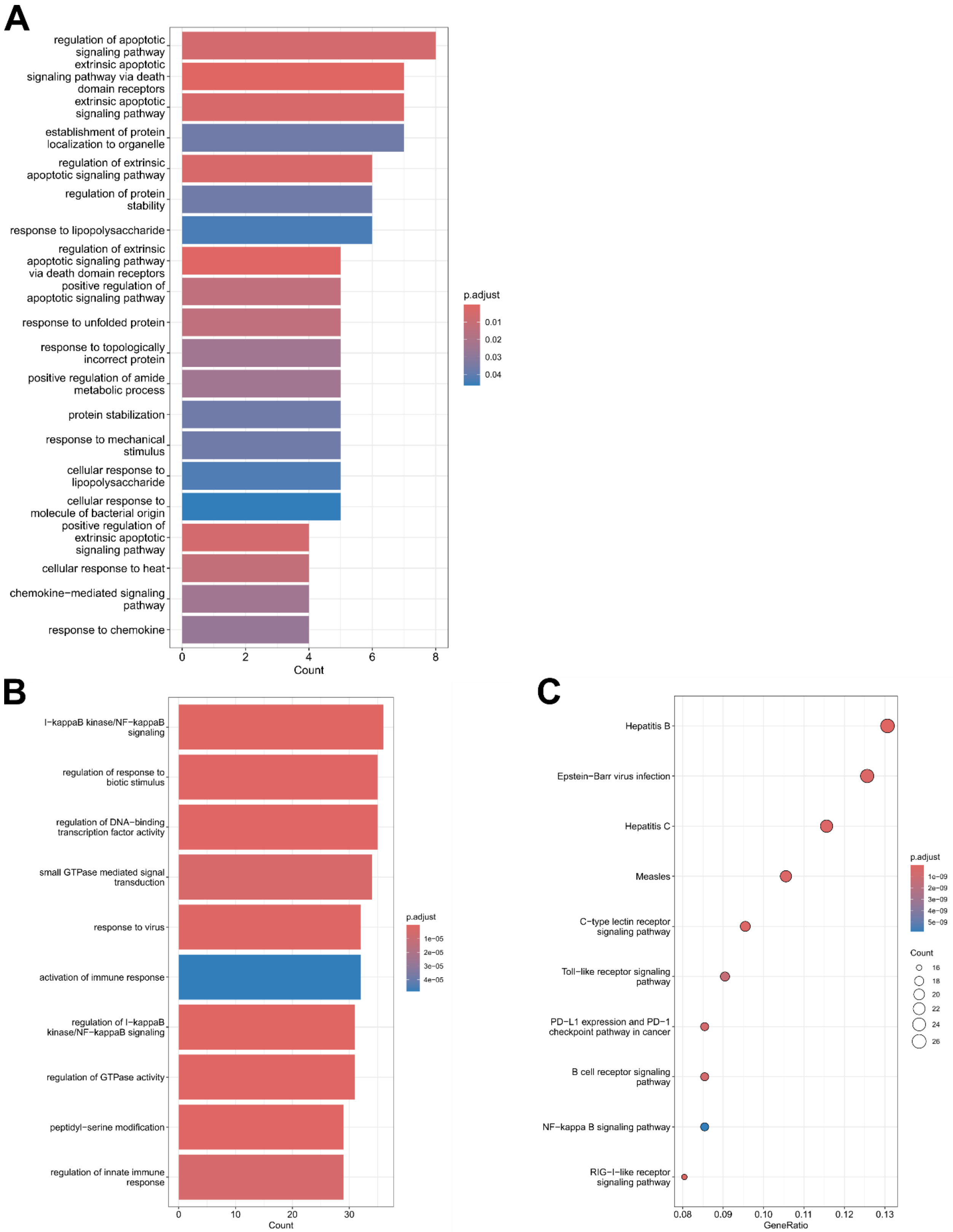

Supplementary Figure 5. Additional pathway enrichment analyses of up- and downregulated proteins identified within preterm infant-derived intestinal epithelial organoids (PIOs) following 3 hr incubation with basolateral flagellin and apical LPS (both 100 ng/ml). **(A)** Gene ontology (GO) biological process terms that were significantly enriched within PIO proteins upregulated during treatment. **(B)** GO biological process terms that were significantly enriched within PIO proteins downregulated during treatment. **(C)** KEGG pathways that were significantly enriched within PIO proteins downregulated during treatment.
